# Synthesis and Antiproliferative Activity of 2,5-bis(3′-Indolyl)pyrroles, Analogues of the Marine Alkaloid Nortopsentin

**DOI:** 10.3390/md11030643

**Published:** 2013-03-01

**Authors:** Anna Carbone, Barbara Parrino, Paola Barraja, Virginia Spanò, Girolamo Cirrincione, Patrizia Diana, Armin Maier, Gerhard Kelter, Heinz-Herbert Fiebig

**Affiliations:** 1 Dipartimento di Scienze e Tecnologie Biologiche, Chimiche e Farmaceutiche, STEBICEF, via Archirafi 32, 90123-Palermo, Italy; E-Mails: anna.carbone@unipa.it (A.C.); barbara.parrino@unipa.it (B.P.); paola.barraja@unipa.it (P.B.); virginia.spano@unipa.it (V.S.); girolamo.cirrincione@unipa.it (G.C.); 2 Oncotest GmbH, Institute for Experimental Oncology, Am Flughafen 12-14, 79108 Freiburg, Germany; E-Mails: armin.maier@oncotest.de (A.M.); gerhard.kelter@oncotest.de (G.K.); heiner.fiebig@oncotest.de (H.-H.F.)

**Keywords:** bis-indolyl-pyrroles, nortopsentin analogues, marine alkaloids, antitumor, *ex-vivo* xenografts

## Abstract

2,5-bis(3′-Indolyl)pyrroles, analogues of the marine alkaloid nortopsentin, were conveniently prepared through a three step procedure in good overall yields. Derivatives **1a** and **1b** exhibited concentration-dependent antitumor activity towards a panel of 42 human tumor cell lines with mean IC_50_ values of 1.54 μM and 0.67 μM, respectively. Investigating human tumor xenografts in an *ex-vivo* clonogenic assay revealed selective antitumor activity, whereas sensitive tumor models were scattered among various tumor histotypes.

## 1. Introduction

**Figure 1:**
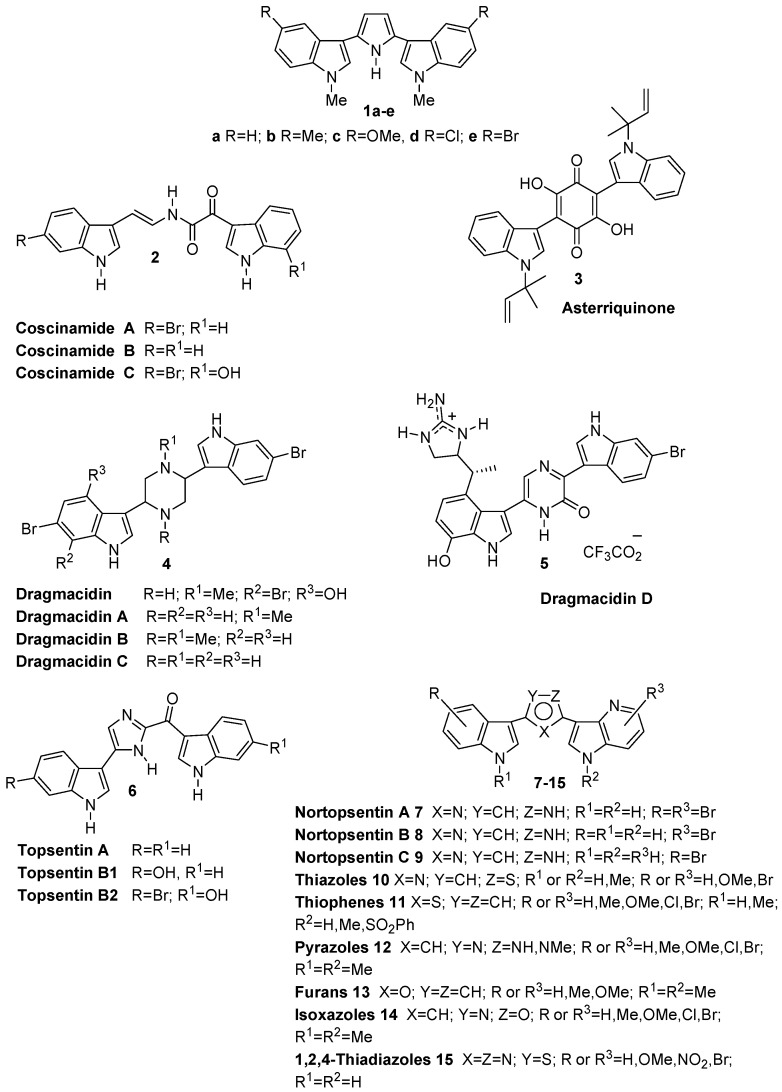
bis-Indolyl alkaloids.

Marine organisms constitute a very important source of biologically active natural products including some of the most potent antineoplastic agents yet discovered [1,2]. In particular, bis-indole alkaloids (Figure 1), characterized by two indole units bound to a spacer through their 3 position, constitute a class of deep-sea sponge metabolites with potent biological activity such as anti-inflammatory, antimicrobial, antiviral and antitumor [3,4,5,6]. Bis-indole alkaloids can bear either an acyclic chain or a six membered carbocyclic or heterocyclic ring or a five membered heterocycle to connect the two indole units. Coscinamides A–C **2**, isolated from deep marine sponge *Coscinoderma* sp. bearing a linear chain as a spacer, showed HIV inhibitory activity [7]. Asterriquinone **3**, isolated from *Aspergillus fungi* and having, as a spacer, a six membered carbocyclic ring showed *in vivo* activity against Ehrlich carcinoma, ascites hepatoma AH13 and mouse P388 leukemia [8]. The first isolated four dragmacidins **4**, containing the six membered heterocyclic link piperazine, were isolated from a large number of deep water sponges including *Dragmacidon*, *Halicortex*, *Spongosorites*, *Hexadella* and the tunicate *Didemnum candidum* and showed, among other biological properties, modest cytotoxic activity [9,10,11]. Successively, more complex components of this family such as dragmacidin D **5**, having a pyrazinone moiety as a spacer, exhibited several biological properties such as inhibition of serine-threonine protein phosphatases, antiviral, antimicrobial and anticancer activities [12,13].

Topsentins **6** isolated from Mediterranean sponge *Topsentia genitrix* exhibited antitumor and antiviral activities [14,15].

Nortopsentins A–C **7**–**9**, bis-indolyl alkaloids having imidazole as a five membered ring spacer, showed *in vitro* cytotoxicity against P388 cells (IC_50_ 4.5–20.7 μM) and their *N*-methylated derivatives showed significant improvement in P388 activity compared to that of the parent compounds (IC_50_ 0.8–2.1 μM) [16,17].

Due to the small amounts of biologically active substances extracted from natural material, several total syntheses of Nortopsentins were proposed [18,19,20,21].

Moreover, due to their interesting biological activities, marine alkaloids are considered to be important lead compounds for the discovery of new biologically active compounds. Thus, dragmacidin analogues, bearing the six membered rings pyridine, pyrimidine, pyrazine and pyrazinone as spacer were synthesized. These analogues showed strong inhibitory activity against a wide range of human tumor cell lines [22,23,24,25].

Nortopsentin analogues bearing five membered heterocycles which replaced the imidazole ring of the natural product were synthesized and exhibited remarkable antiproliferative activity, often reaching IC_50_ values at sub-micromolar level. Thus bis-indolyl-thiazoles **10** [22,26], thiophenes **11** [27], pyrazoles **12** [28], furans **13** [29], isoxazoles **14** [29], and 1,2,4-thiadiazoles **15**, were reported [30]. Moreover, in other bis-indolyl analogues, which—apart from the heterocyclic spacer—were also modified, one or both indole units have been described. In particular, 3-indolyl-5-phenylpyridine showed antiproliferative activity in the range 5–15 μM and inhibited CDK1 at 0.3–0.7 μM level [31]; phenylthiazolyl-7-azaindoles showed antiproliferative activity against a wide range of human tumor cell lines at micromolar to nanomolar concentrations and inhibited CDK1 with IC_50_ values in the range 0.41–0.85 μM [32].

In this paper we report the synthesis of substituted 2,5-bis(3′-indolyl)pyrroles of type **1**, nortopsentin analogues in which the imidazole ring spacer of the natural product is replaced by the pyrrole ring. Furthermore, antitumor activity of the new nortopsentin analogues was investigated by an *in vitro* cytotoxicity assay using human tumor cell lines and an *ex-vivo* clonogenic assay using human tumor xenograft. 

## 2. Results and Discussion

**Scheme 1:**
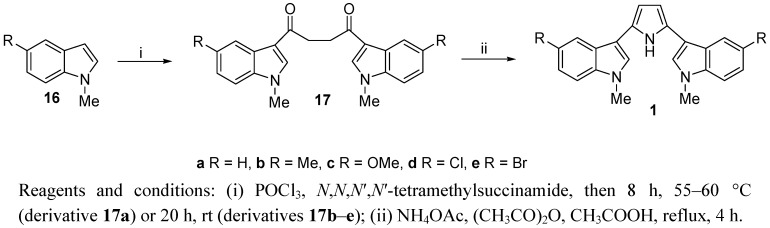
Synthesis of substituted 2,5-bis(3′-indolyl)pyrroles **1a**–**e**.

A general synthesis of 2,5-bis(3′-indolyl)pyrroles **1a**–**e** is shown in Scheme 1. *N*-methyl indoles **16a**–**e** were transformed into 1,4-butanediones **17a**–**e** in good yields by a Vilsmeier-Haack reaction with phosphorus oxychloride and tetramethylsuccinamide [29]. 1,4-Diketones **17a**–**c** were purified by flash chromatography whereas 1,4-diketones **17d**,**e** resulted unstable and were used for the next step as crude products. All symmetrical 1,4-diketones **17** were converted into the corresponding 2,5-bis(3′-indolyl)pyrroles **1a**–**e** using ammonium acetate, acetic anhydride in acetic acid under reflux [33].

By using a monolayer cell survival and proliferation assay the five bis-indolyl-pyrroles **1a**–**e** were screened for *in vitro* antitumor activity in a panel of 12 human tumor cell lines. All compounds showed cytotoxic activity in at least the highest test concentration of 100 μg/mL, exhibiting mean IC_50_ values in the range from 4.4 μg/mL to 0.37 μg/mL (Table 1). Adriamycin tested in parallel was used as cytotoxic positive control and showed concentration-dependent anti-cancer activity towards all cell lines. 

**Table 1:** *In vitro* activity of derivatives **1a**–**e** towards 12 human cell lines.

Compound	IC_50_ (μg/mL)	Active/Total ^a^			Tumor Selectivity ^b,c^	
		1 (μg/mL)	10 (μg/mL)	100 (μg/mL)	A	B
**1a**	0.37	8/12 (67%)	12/12 (100%)	12/12 (100%)	1/12	+
**1b**	0.37	8/12 (67%)	12/12 (100%)	12/12 (100%)	1/12	+
**1c**	3.4	0/12 (0%)	10/12 (83%)	12/12 (100%)	0/12	−
**1d**	3.4	1/12 (8%)	9/12 (75%)	12/12 (100%)	1712	+
**1e**	4.4	0/12 (0%)	9/12 (75%)	12/12 (100%)	0/12	−
Adr ^d^	0.007	4/12 (33%)	10/12 (83%)	11/12 (92%)	2/12	++

^a^ Responsive (T/C < 30%)/total cell lines. ^b^ A = selective (individual IC70 < 1/3 mean IC70)/total cell lines. ^c^ B = rating, − (0/10 selective), + (1/10 selective), ++ (2/10 selective), +++ (≥3/10 selective). ^d^ Adr = Adriamycin, active/total is given at 0.03, 0.3, and 3 μg/mL.

The most active candidates **1a** and **1b** were further profiled in monolayer cultures of 42 human tumor cell lines, reflecting 15 different solid tumor types (Table 2A). 

Compounds **1a** and **1b** effected concentration-dependent inhibition of tumor cell growth with mean IC_50_ values of 1.54 μM and 0.67 μM, indicating pronounced cytotoxic potency. 

Regarding compound **1a**, selective activity (as defined by individual IC_50_ value of a distinct cell line <1/2 mean IC_50_ value over the 42 cell lines) was detected in two out of three cell lines of bladder cancer (BXF 1218L, BXF 1352L), two out of three melanoma cell lines (MEXF 1341L; MEXF 276L), as well as in the cell lines LXFL 1121L (lung cancer), PAXF PANC-1 (pancreatic cancer), SXF SAOS-2 (sarcoma) and UXF 1138L (cancer of the uterine body). Particular less sensitive cell lines were found among colon (HCT-116, HT-29), lung (LXFA 289L), ovarian (OVXF 899L), prostate (DU145) and renal cancer (RXF 393NL, RXF 486L). 

Compound **1b** exhibited pronounced activity towards cell lines derived from bladder cancer (BXF 1218L), melanoma (MEXF 1341L, MEXF 276L), prostate cancer (PRXF PC3M) and sarcoma (SXF SAOS-2). 

**Table 2:** *In vitro* and *ex vivo* anti-tumor activity judged by IC_50_ values (μM). (**A**) *In vitro* tumor cell lines (monolayer assay); (**B**) *Ex-vivo* human xenografts (clonogenic assay).

Cell line					Tumor			
histotype	name		1a	1b	histotype	name	1a	1b
**Bladder**	**BXF**	**1218L**	0.72	0.32	**Bladder**	**BXF 1218**	2.45	2.35
	**BXF**	**1352L**	0.68	0.41		**BXF 1228**	2.89	2.86
	**BXF**	**T24**	1.72	0.58	**Colon**	**CXF 1103**	4.47	18.36
**Colon**	**CXF**	**269L**	1.39	0.56		**CXF 1729**	3.49	7.77
	**CXF**	**HCT116**	3.24	1.64		**CXF 1783**	37.57	40.70
	**CXF**	**HT29**	5.20	2.65		**CXF 280**	26.18	35.70
	**CXF**	**RKO**	1.50	0.63		**CXF 975**	6.93	6.03
**Gastric**	**GXA**	**MKN45**	0.84	0.53	**Gastric**	**GXF 1172**	6.27	>100
	**GXF**	**251L**	1.56	0.65		**GXF 251**	7.10	25.50
**Head&Neck**	**HNXF**	**CAL27**	0.81	0.50		**GXF 97**	2.72	3.74
**Liver**	**LIXF**	**575L**	1.84	0.61	**Head&Neck**	**HNXF 536**	2.45	2.67
**Lung**	**LXFA**	**289L**	6.34	2.39		**HNXF 908**	2.57	4.00
	**LXFA**	**526L**	1.57	0.67	**Lung**	**LXFA 1012**	4.76	27.34
	**LXFA**	**629L**	2.28	1.36		**LXFA 1584**	2.07	2.73
	**LXFL**	**1121L**	0.67	0.38		**LXFA 297**	54.90	>100
	**LXFL**	**529L**	1.85	0.67		**LXFA 526**	2.96	3.15
	**LXFL**	**H460**	2.08	0.89		**LXFA 629**	2.23	3.63
**Mammary**	**MAXF**	**401NL**	1.24	0.64		**LXFA 677**	23.19	27.02
	**MAXF**	**MCF7**	2.44	0.98		**LXFA 923**	19.50	25.68
	**MAXF**	**MDA231**	1.18	0.49		**LXFE 1422**	5.90	1.72
**Melanoma**	**MEXF**	**1341L**	0.52	0.19		**LXFL 1072**	3.44	3.42
	**MEXF**	**276L**	0.22	0.11		**LXFL 529**	5.07	4.46
	**MEXF**	**462NL**	1.31	0.55		**LXFL 625**	24.04	38.21
**Ovarian**	**OVXF**	**OVCAR3**	1.46	0.58	**Mammary**	**MAXF 1322**	16.80	9.49
	**OVXF**	**899L**	5.40	2.03		**MAXF 1384**	34.48	33.28
**Pancreatic**	**PAXF**	**PANC1**	0.74	0.41		**MAXF 401**	5.90	11.32
	**PAXF**	**1657L**	2.68	1.02	**Melanoma**	**MEXF 1539**	19.90	15.31
	**PAXF**	**546L**	2.70	1.16		**MEXF 276**	1.44	1.75
**Prostate**	**PRXF**	**22RV1**	1.46	0.63		**MEXF 462**	3.24	4.10
	**PRXF**	**DU145**	4.45	1.96		**MEXF 989**	1.18	0.58
	**PRXF**	**LNCAP**	2.10	0.68	**Ovarian**	**OVXF 1353**	20.96	22.90
	**PRXF**	**PC3M**	0.85	0.32		**OVXF 899**	3.54	3.93
**Plerameso-**	**PXF**	**1118L**	2.50	0.88	**Pancreatic**	**PAXF 546**	3.52	14.63
**thelioma**	**PXF**	**1752L**	0.89	0.43		**PAXF 736**	2.99	2.10
	**PXF**	**698L**	1.86	0.86	**Prostate**	**PRXF DU145**	28.67	25.98
**Renal**	**RXF**	**1183L**	1.13	0.58		**PRXF PC3M**	2.89	2.80
	**RXF**	**1781L**	1.77	0.66	**Pleurameso-**	**PXF 1752L**	3.71	4.05
	**RXF**	**393NL**	3.14	1.34	**thelioma**	**PXF 541**	2.20	0.37
	**RXF**	**486L**	3.86	1.60	**Renal**	**RXF 1220**	6.34	3.16
**Sarcoma**	**SXF**	**SAOS2**	0.72	0.33		**RXF 486**	2.90	3.90
	**SXF**	**TE671**	1.60	0.53		**RXF 631**	2.98	2.81
**Uterus**	**UXF**	**1138L**	0.72	0.35	**Sarcoma**	**SXF 1186**	5.71	6.17
						**SXF 1301**	3.54	23.95
						**SXF 627**	3.40	4.06
**geometric mean IC_50_**			**1.54**	**0.67**	**geometric mean IC_50_**		**5.69**	**7.25**
**Tumor selectivity ^1)^**			**8/42**	**5/42**	**Tumor selectivity ^1)^**		**9/44**	**14/44**
**(selective/total)**			**(19%)**	**(12%)**	**(selective/total)**		**(20%)**	**(32%)**
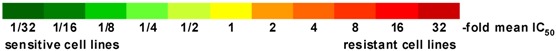								

^1)^ Number of cell lines/xenografts with IC_50_ < 1/2 (mean IC_50_)/total.

Inhibition of clonogenicity of tumor cells was evaluated in additional tumor models using an *ex vivo* clonogenic assay (Table 2B). The anti-proliferative activity of **1a** and **1b** was evaluated in cell suspensions prepared from 44 human tumor xenografts of 13 different tumor types, which were cultured as solid tumors in serial passage on immune deficient nude mice. The results confirmed the concentration-dependent activity of **1a** and **1b** on cell lines with mean IC_50_ values of 5.69 μM (**1a**) and 7.25 μM (**1b**), respectively. With regard to **1a**, IC_50_ values ranged from 1.18 μM to 54.9 μM, corresponding to a 46-fold difference. Selective activity was found against 9 out of the 44 tumors tested, while these sensitive tumors were scattered among various tumor histotypes, like bladder, gastric, head and neck, and lung cancer, as well as melanoma and pleuramesothelioma. 

Pronounced tumor selectivity was found for compound **1b**, with 14 out of 44 tumors (32%) showing IC_50_ values <3.6 μM (=1/2 mean IC_50_ value). IC_50_ values for **1b** ranged from 0.37 μM (PXF 541) to >100 μM (GXF 1172, LXFA 297), equivalent to more than 270-fold difference between resistant and sensitive tumor models. Sensitive tumors were found among bladder, head and neck, lung, pancreatic, prostate and renal cancer as well as melanoma and pleuromesothelioma.

Cells that show anchorage independent growth in semi-solid medium contain, to a certain extent, tumor stem cells which are considered to be responsible for the metastatic and infiltrative potential of a tumor [34,35,36,37]. Thus, the clonogenic assay may inter alia be used to identify candidate tumors for subsequent *in vivo* studies [34,35,38,39,40]. First *in vivo* efficacy studies, using the patient-derived melanoma explants MEXF 276 and MEXF 989, did not result in tumor growth inhibition (data not shown). Further *in vivo* efficacy studies of candidate tumors as selected by the results of the clonogenic assay may be warranted. 

## 3. Experimental Section

### 3.1. Chemistry

#### 3.1.1. General Procedure

All the commercially available reagents and solvents were used without further purification. 1,2-Diaza-1,3-diene (DD) **13** was synthesized as a mixture of *E/Z* isomers as previously reported [15,16]. Column chromatography was performed with Merck silica gel 230–400 Mesh ASTM or with Büchi Sepacor chromatography module (prepacked cartridge system). TLC analysis was performed on pre-loaded (0.25 mm) glass supported silica gel plates (Kieselgel 60); compounds were visualized by exposure to UV light and by dipping the plates in 1% Ce(SO_4_)·4H_2_O, 2.5% (NH_4_)_6_Mo_7_O_24_·4H_2_O in 10% sulfuric acid followed by heating on a hot plate. ^1^H NMR and ^13^C NMR spectra were recorded in DMSO-*d*_6_ solution on 200 (Bruker AC) MHz instrument. Proton and carbon spectra were referenced internally to solvent signals, using values of δ = 2.49 ppm for proton (middle peak) and δ = 39.50 ppm for carbon (middle peak) in DMSO-*d*_6_. The following abbreviations are used to describe peak patterns where appropriate: s = singlet, d = doublet, t = triplet, q = quartet and m = multiplet. All coupling constants (*J*) are given in Hz. All melting points were taken on a Büchi-Tottoli capillary apparatus and are uncorrected; IR spectra were determined in bromoform or nujol with a Jasco FT/IR 5300 spectrophotometer. Mass spectra were recorded in the EI mode (70 eV) on a Shimadzu GC-MS QP5050A spectrometer. Elemental analyses (C, H, N) were within ±0.4% of the theoretical values.

#### 3.1.2. Synthesis of *N,N,N′,N′*-Tetramethylsuccinamide

5.3 mL (0.05 mol) of succinyl chloride at 0 °C was added dropwise to a solution of dimethylamine (40% in water, 2 mmol). The mixture was stirred for 30 min and then extracted with DCM, dried and evaporated to afford the pure *N*,*N*,*N′*,*N′*-tetramethylsuccinamide. Analytical and spectroscopic data are reported elsewhere [21].

#### 3.1.3. General Procedure for the Preparation of 1,4-bis(Indol-3-yl)butane-1,4-diones (**17a–e**)

Phosphorus oxychloride (5.3 mL, 57 mmol) was slowly added to *N*,*N*,*N′*,*N′*-tetramethylsuccinamide (2.58 g, 15 mmol) at 10–20 °C and the mixture was stirred for 24 h. Then *N*-methylindoles **16a**–**e** (30 mmol) were slowly added keeping the temperature below 45 °C. After the addition was complete the mixture was heated for 8 h to 55–60 °C (for derivative **17a**) or stirred at rt for 20 h (for derivatives **17b**–**e**). The solution was poured onto crushed ice, made basic with sodium hydroxide 10 M and filtered. The solid was washed with water, dried and purified by chromatography using (DCM/ethyl acetate 9/1) as eluent to afford the pure derivatives **17a**–**c**; whereas derivatives **17d**,**e** were used for next step without purification. Analytical and spectroscopic data are reported elsewhere [29].

#### 3.1.4. General Procedure for the Preparation of Pyrroles (**1a–e**)

Butanediones **17a**–**e** (2 mmol) was refluxed under N_2_ for 4 h with ammonium acetate (3.93 g, 51 mmol) and acetic anhydride (1.6 mL, 16.9 mmol) in acetic acid (20 mL). The solution was poured into ice water, and the solid obtained was filtered, dried and purified by chromatography using dichloromethane as eluent.

##### 3.1.4.1. 3,3′-(1*H*-Pyrrole-2,5-diyl)bis(1-methyl-1*H*-indole) (**1a**)

Green solid; yield: 65%; mp: 175 °C; IR 3438 (NH) cm^−1^; ^1^H NMR (200 MHz, DMSO-*d*_6_) δ: 3.82 (s, 3H), 6.46 (s, 1H), 7.12 (t, 1H, *J* = 7.2 Hz), 7.21 (t, 1H, *J* = 7.2 Hz), 7.45 (d, 1H, *J* = 7.6 Hz), 7.68 (s, 1H), 7.89 (d, 1H, *J* = 7.6 Hz), 10.80 (s, 1H); ^13^C NMR (50.3 MHz, DMSO-*d*_6_) δ: 32.5 (CH_3_), 105.3 (CH), 108.7 (C), 109.8 (CH), 119.2 (CH), 119.9 (CH), 121.4 (CH), 125.0 (C), 125.1 (CH), 125.8 (C), 136.8 (C). Anal. Calcd for C_22_H_19_N_3_: C, 81.20; H, 5.89; N, 12.91. Found: C, 80.94; H, 5.53; N, 13.22.

##### 3.1.4.2. 3,3′-(1*H*-Pyrrole-2,5-diyl)bis(1,5-dimethyl-1*H*-indole) (**1b**)

Green solid; yield: 60%; mp: 177–178 °C; IR 3454 (NH) cm^−1^; ^1^H NMR (200 MHz, DMSO-*d*_6_) δ: 2.45 (s, 3H), 3.80 (s, 3H), 6.43 (s, 1H), 7.03 (d, 1H, *J* = 8.4 Hz), 7.34 (d, 1H, *J* = 8.4 Hz), 7.61 (s, 1H), 7.67 (s, 1H), 10.80 (s, 1H); ^13^C NMR (50.3 MHz, DMSO-*d*_6_) δ: 21.3 (CH_3_), 32.5 (CH_3_), 105.2 (CH), 108.2 (C), 109.5 (CH), 119.5 (CH), 123.0 (CH), 125.2 (CH), 125.3 (C), 125.8 (C), 127.7 (C), 135.3 (C). Anal. Calcd for C_24_H_23_N_3_: C, 81.55; H, 6.56; N, 11.89. Found: C, 81.26; H, 6.28; N, 11.60. 

##### 3.1.4.3. 3,3′-(1*H*-Pyrrole-2,5-diyl)bis(5-methoxy-1-methyl-1*H*-indole) (**1c**)

Green solid; yield: 60%; mp: 148–150 °C; IR 3452 (NH) cm^−1^; ^1^H NMR (200 MHz, DMSO-*d*_6_) δ: 3.80 (s, 3H), 3.83 (s, 3H), 6.41 (s, 1H), 6.86 (dd, 1H, *J* = 2.4, 9.0 Hz), 7.30 (d, 1H, *J* = 2.4 Hz), 7.37 (d, 1H, *J* = 9.0 Hz), 7.60 (s, 1H), 10.80 (s, 1H); ^13^C NMR (50.3 MHz, DMSO-*d*_6_) δ: 32.7 (CH_3_), 55.4 (CH_3_), 101.6 (CH), 105.1 (CH), 108.3 (C), 110.6 (CH), 111.4 (CH), 125.3 (C), 125.7 (C), 125.8 (CH), 132.1 (C), 153.7 (C). Anal. Calcd for C_24_H_23_N_3_O_2_: C, 74.78; H, 6.01; N, 10.90. Found: C, 74.99; H, 5.75; N, 11.14.

##### 3.1.4.4. 3,3′-(1*H*-Pyrrole-2,5-diyl)bis(5-chloro-1-methyl-1*H*-indole) (**1d**)

Green solid; yield: 50%; mp: 159–160 °C; IR 3444 (NH) cm^−1^; ^1^H NMR (200 MHz, DMSO-*d*_6_) δ: 3.84 (s, 3H), 6.42 (s, 1H), 7.21 (d, 1H, *J* = 8.6 Hz), 7.51 (d, 1H, *J* = 8.6 Hz), 7.72 (s, 1H), 7.83 (s, 1H), 10.90 (s, 1H); ^13^C NMR (50.3 MHz, DMSO-*d*_6_) δ: 32.7 (CH_3_), 105.7 (CH), 108.4 (C), 111.5 (CH), 118.9 (CH), 121.3 (CH), 124.1 (C), 125.2 (C), 125.9 (C), 127.0 (CH), 135.3 (C). Anal. Calcd for C_22_H_17_Cl_2_N_3_: C, 67.01; H, 4.35; N, 10.66. Found: C, 66.77; H, 4.08; N, 10.37.

##### 3.1.4.5. 3,3′-(1*H*-Pyrrole-2,5-diyl)bis(5-bromo-1-methyl-1*H*-indole) (**1e**)

Green solid; yield: 50%; mp: 137 °C; IR 3446 (NH) cm^−1^; ^1^H NMR (200 MHz, DMSO-*d*_6_) δ: 3.84 (s, 3H), 6.41 (s, 1H), 7.32 (dd, 1H, *J* = 1.9, 8.6 Hz), 7.48 (d, 1H, *J* = 8.6 Hz), 7.70 (s, 1H), 7.97 (d, 1H, *J* = 1.9 Hz), 11.00 (s, 1H); ^13^C NMR (50.3 MHz, DMSO-*d*_6_) δ: 32.7 (CH_3_), 105.8 (CH), 108.3 (C), 112.0 (CH), 112.0 (C), 121.9 (CH), 123.9 (CH), 125.2 (C), 126.6 (C), 126.9 (CH), 135.5 (C). Anal. Calcd for C_22_H_17_Br_2_N_3_: C, 54.68; H, 3.55; N, 8.70. Found: C, 54.48; H, 3.45; N, 8.33.

### 3.2. Biology

#### 3.2.1. *In Vitro* Antitumor Activity towards Permanent Growing Human Tumor Cell Lines

Antitumor activity of the compounds was tested in a monolayer cell survival and proliferation assay using human tumor cell lines. Studies using panels of human tumor cell lines of different origin/histotype allow for analysis of potency and tumor selectivity of test compounds and to identify active compounds that qualify for further preclinical evaluation.

##### 3.2.1.1. Cell Lines

24 out of the 42 cell lines as tested were established at Oncotest from patient-derived human tumor xenografts passaged subcutaneously in nude mice [41]. The origin of the donor xenografts was described [34,42]. The other cell lines were obtained from ATCC (Rockville, MD, USA), DSMZ (Braunschweig, Germany) were kindly provided by the National Cancer Institute (Bethesda, MA, USA). Cells were cultured in RPMI 1640 medium, supplemented with 10% fetal calf serum and 0.1 mg/mL gentamicin under standard conditions (37 °C, 5% CO_2_). Authenticity of all cell lines was proven by STR analysis at the DSMZ.

##### 3.2.1.2. Cytotoxicity Assay (Monolayer Assay)

A modified propidium iodide assay was used to assess the compounds’ activity toward human tumor cell lines [29]. Briefly, cells were harvested from exponential phase cultures by trypsinization, counted and plated in 96-well flat-bottom microtiter plates at a cell density dependent on the cell line (4000–20,000 cells/well). After 24 h recovery period to allow the cells to adhere and resume exponential growth, test compounds were added at 10 concentrations in half-log increments and left for further 4 days. The inhibition of proliferation was determined by measuring the DNA content using an aqueous propidium iodide solution (7 μg/mL). Fluorescence was measured using the Cytofluor micro-plate reader (excitation λ = 530 nm, emission λ = 620 nm), providing a direct relationship to the total viable cell number. In each experiment, all data points were determined in triplicates. Relative IC_50_ values were determined by non-linear regression using the analysis software GraphPad Prism^®^ (GraphPad software Inc., La Jolla, CA, USA).

#### 3.2.2. *Ex-Vivo* Antitumor Activity towards Tumor Xenografts

Effects of the test compounds on clonogenicity of tumor cells were investigated in a clonogenic assay. Tumor xenografts were derived from patient tumors engrafted as a subcutaneously growing tumor in NMRI nu/nu mice obtained from Oncotest’s breeding facility [35,36]. Details of the test procedure have been described earlier [38]. Briefly, solid human tumor xenografts were removed from mice under sterile conditions, mechanically disaggregated and subsequently incubated with an enzyme cocktail consisting of collagenase type IV (41 U/mL), DNase I (125 U/mL), hyaluronidase type III (100 U/mL) and dispase II (1.0 U/mL) in RPMI 1640-Medium at 37 °C for 60 min. Cells were passed through sieves of 200 μm and 50 μm mesh size and washed twice with sterile PBS-buffer. The percentage of viable cells was determined in a Neubauer-hemocytometer using trypan blue exclusion. The assay contained 3 layers of equal volume. The bottom layer consisted of 0.2 mL/well Iscove’s Modified Dulbecco’s Medium (IMDM, Life Technologies), supplemented with 20% (v/v) fetal calf serum (Sigma), 0.01% (w/v) gentamicin (Life Technologies) and 0.75% (w/v) agar (BD Biosciences). 1.5 × 10^4^ to 4 × 10^4^ cells were added to 0.2 mL of the same culture medium supplemented with 0.4% (w/v) agar and plated in 24-multiwell dishes onto the bottom layer. The test compounds were applied by continuous exposure (drug overlay) in 0.2 mL of culture medium. Every dish included six untreated control wells and drug-treated groups in triplicate at 6 concentrations. Cultures were incubated at 37 °C and 7.5% CO_2_ in a humidified atmosphere for 7–20 days and monitored closely for colony growth using an inverted microscope. Within this period, *in vitro* tumor growth led to the formation of colonies with a diameter of >50 μm. At the time of maximum colony formation, counts were performed with an automatic image analysis system (Bioreader 5000-Wα, Biosys GmbH). 24 h prior to evaluation, vital colonies were stained with a sterile aqueous solution of 2-(4-iodophenyl)-3-(4-nitrophenyl)-5-phenyltetrazolium chloride (1 mg/mL, 100 μL/well). Relative IC_50_ values were calculated as described in 3.2.1.2.

## 4. Conclusions

In the present study, the synthesis and characterization of five substituted 2,5-bis(3′-indolyl)pyrroles of type **1**, nortopsentin analogues, in which the imidazole ring spacer of the natural product is replaced by the pyrrole ring, was described. Among them, **1a** and **1b** showed antitumor activity in the low micromolar or even sub-micromolar range towards a panel of human tumor cell lines *in vitro*. Furthermore, in the *ex-vivo* clonogenic assay, pronounced tumor selectivity was detected, in particular for **1b**, whereas sensitive tumors were scattered among various tumor histotypes.
